# Isometric Force–Time Curve Assessment: Accuracy, Precision, and Repeatability of a Mobile Application and Portable and Lightweight Device

**DOI:** 10.3390/sports12100268

**Published:** 2024-10-07

**Authors:** Dario Santos, Alfredo Bravo-Sánchez, Leonardo Alexandre Peyré-Tartaruga, Franco Simini, Rodrigo Zacca

**Affiliations:** 1 Unidad Académica de Fisioterapia, Facultad de Medicina, Universidad de la República, Montevideo 11600, Uruguay; santosdario69@gmail.com; 2 Núcleo de Ingeniería Biomédica (NIB), Universidad de la República, Montevideo 11600, Uruguay; simini@fing.edu.uy; 3 Faculty of Health Sciences, Universidad Francisco de Vitoria, 28223 Pozuelo de Alarcón, Spain; alfredo.bravo@ufv.es; 4 Performance and Sport Rehabilitation Laboratory, Faculty of Sport Sciences, University of Castilla-La Mancha, 45071 Toledo, Spain; 5 Biodynamics Laboratory (LaBiodin), Universidade Federal do Rio Grande do Sul, Porto Alegre 90690-200, Brazil; leonardo.tartaruga@ufrgs.br; 6 Human Locomotion Laboratory (LocoLab), Department of Public Health, Experimental Medicine and Forensic Sciences, University of Pavia, 27100 Pavia, Italy; 7 Research Center in Physical Activity, Health and Leisure (CIAFEL), Faculty of Sports, University of Porto (FADEUP), 4200-450 Porto, Portugal; 8 Laboratory for Integrative and Translational Research in Population Health (ITR), 4050-600 Porto, Portugal

**Keywords:** exercise, health, physical fitness, rehabilitation, physical therapy, strength assessment, exercise physiology, sports biomechanics, digital technology

## Abstract

Strength assessment is one of the main fields in sports performance, physical rehabilitation, physical activity, and health. We aimed to compare maximal voluntary isometric contractions (MVICs) and paired voluntary isometric contractions (VICs) of knee extensors between an isokinetic dynamometer (BIODEX) and a portable and lightweight device (DINABANG). From 19 volunteers (age: 28.7 ± 7 years; body mass: 72 ± 10 kg; and height: 173 ± 7 cm) we obtained 114 paired MVIC measures and, from the force–time curves of these repetitions, 22,507 paired VIC measures of knee extensors. We observed “excellent” repeatability for MVICs (ICC:1.00; *p* < 0.001) between BIODEX (247 ± 79.5 Nm) and DINABANG (247 ± 74.8 Nm), with “trivial” effect (mean difference: 0.12 Nm (0.02%); 95%CI: −0.13 to 0.23 Nm; *p* = 0.606; d = 0.048). Bland–Altman plots revealed high accuracy for MVIC (bias: 0.12 Nm) and consistent distribution (precision) inside the limits of agreement (−4.81 to 5.06 Nm) and respective 95%CI. “Excellent” repeatability was also observed for VICs (ICC:1.00; *p* < 0.001) between BIODEX (219 ± 84.1 Nm) and DINABANG (218 ± 84.0 Nm), with “trivial” effect (0.24 Nm (0.11%); 0.08 to 0.11 Nm; *p* < 0.001; d = 0.100). Bland–Altman plots revealed high accuracy for VICs (bias: 0.24 Nm) and consistent distribution (precision) inside the limits of agreement (−4.5 to 4.9 Nm) and respective 95%CI. DINABANG is accurate, precise, and reliable in torque measurement.

## 1. Introduction

Muscle strength assessments are critical in rehabilitation, sports training, physical activity, and health centers [1,2]. A crucial concern in sport sciences and other research area is achieving a reliable and reproducible measurement. Many different types of equipment have been used in such assessments (e.g., simple or isokinetic dynamometers), and the focus has been on different populations, such as athletes or patients [3,4]. In most of these studies, the measurement of maximal isometric strength has been associated with improved sports performance [5], a reduction in the risk of falls in older people [6], or a sign of progress during the rehabilitation process [4]. Therefore, accurate and valid measurement systems are needed to reduce the degree of uncertainty to a minimum.

Isokinetic dynamometry represents the clinical “gold standard” for strength assessment, but it is only available in a limited number of specialized laboratories. In fact, isokinetic dynamometry has some limitations: it lacks portability, takes up a lot of physical space, requires a high amount of training time for its use, and is expensive for some professionals. In response to these limitations, some authors have used their own dynamometers, which are cheaper, portable, and validated [7]. Despite that, most of these devices only measure forces in the perpendicular direction, which limits their application to more realistic gestures [8]. In this direction, after a knee injury, measurements of maximal isometric strength of the hamstrings and quadriceps provide important information for clinicians to help in making the decision on whether the athlete is fit to participate in physical activities [5]. Objective measurements of muscle strength become essential in knowing how the patient’s treatment is progressing.

Typically, during a lower limb exercise program, the investigator/professional defines and controls the subject’s effort based on subjective criteria until a quantitative assessment (e.g., isokinetic or static strength assessment) is required [9]. Specifically, when elastic bands are used to exercise the lower extremities, the question remains as to whether the force deployed is excessive or insufficient, with both situations being suboptimal due to the risk of injury or lack of effectiveness [10]. Therefore, it is necessary to use systems that accurately assess the torque performed by the athlete or patient, to optimize training. The DINABANG mobile application and its portable and lightweight device (Movi, https://www.movi-ing.com/ accessed on 12 May 2024) for isometric force–time curve assessment could address this problem by measuring, in real time, the torque of the involved limb, to guide the decisions of the physiotherapist, the instructed patient, or the athlete during rehabilitation and training sessions. This system has already been validated in some preliminary studies comparing different instruments [11,12].

A recent systematic review revealed that some current apps used in rehabilitation and exercise setups are valid and reliable for measuring bar movement velocity during lower and upper body resistance exercises; however, a significant systematic bias was detected with heavier loads [13]. Although many low-cost dynamometers have recently been validated [13,14], strength devices coupled with angular position measurement are still lacking. DINABANG could address this problem by measuring, in real time, the lower limb torque, to guide the decisions of the physiotherapist, the instructed patient, or the athlete during rehabilitation or training sessions. DINABANG was designed with a strain gauge affixed between the elastic band and a brace just above the malleoli, an inertial measurement unit (IMU), and Bluetooth communication [15]. Thus, the aim of this study was to compare knee joint maximal isometric force–time-curve-related variables between the gold standard (isokinetic dynamometer) BIODEX (BIODEX System 4 Pro: BIODEX Medical Systems, Shirley, NY, USA) and DINABANG by verifying the accuracy, precision, and repeatability. We hypothesized that the knee joint maximal isometric force–time curves between BIODEX and DINABANG are comparable.

## 2. Materials and Methods

We performed an experiment to simultaneously measure torque with DINABANG and BIODEX during standard exercise. DINABANG is a wearable device that tracks force, angle, speed, and torque during exercise. In addition to measuring force using a load cell (like conventional dynamometers), it uses accelerometers and gyroscopes to measure the patient’s position and the angle of action of the force in real time, allowing it to calculate the tangential force and torque during exercise. The data are sent via Bluetooth to a mobile application, which stores the information in a protected database. The exercise protocol consisted of a maximum voluntary isometric contraction (MVIC) of knee extensors, with knee flexed at 60º. Nineteen volunteers (age 28.7 ± 7 years, weight, 72 ± 10 kg, height: 173 ± 7 cm, BMI: 23.8 ± 2.4 kg/cm^2^) participated in the study. All subjects were healthy, with no prior history of cardiovascular, neuromuscular, or orthopedic disease. At the time of inclusion in the study, participants had not engaged into any systematic physical training program within the last year. The volunteers practiced some form of physical activity at least three times a week for at least half an hour per session (Tegner activity level ≥ 5) [12]. Participants were encouraged to avoid extenuating exercise in the 48 h before the study. All subjects agreed to participate voluntarily and signed a written informed consent prior to enrollment. The study protocol was approved by the Research Ethics Committee of the of Faculty of Sports—University of Porto (CEFADE 30_2022; Date of Approval: 16 March 2023).

### 2.1. Calibration

The MVIC of knee extensors was performed using BIODEX (BIODEX System 4 Pro: BIODEX Medical Systems, Shirley, NY, USA) with a sampling frequency of 100 Hz. Simultaneous measurements were made using DINABANG with a sampling frequency of 200 Hz, decimated down to 100 Hz for compatibility with BIODEX. The calibration of BIODEX was regularly checked according to the manufacturer’s specifications. DINABANG has a factory calibration, which was performed by establishing a calibration curve using standard reference masses. https://in.mathworks.com (accessed on 12 May 2024).

### 2.2. Study Design and Protocol

**Figure 1:**
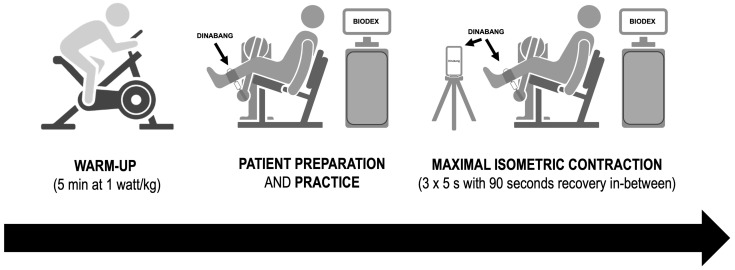
DINABANG and BIODEX setup for simultaneous lower limb force measurements.

The study design consisted of a warm-up (5 min cycling at 1 Watt/kg), familiarization, and practice with the equipment and the test protocol (Figure 1).

**Figure 2:**
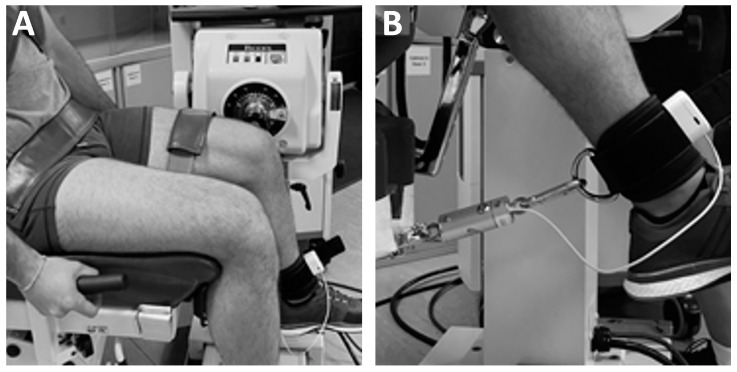
Maximum voluntary isometric contraction (MVIC) of knee extensor setup and DINABANG connection. (**A**) Volunteer sitting in BIODEX chair with DINABANG strap connected just above the malleolus in lieu of the usual BIODEX cylinder, (**B**) detail of DINABANG connected both to the ankle strap and to the BIODEX force connection at the back. Note: DINABANG includes a carabine hook secured to the ankle strap.

The subjects performed three repetitions of a MVIC of knee extensors with each lower limb (6 repetitions in total). After each repetition, the patient was given at least 90 s of rest and the process of placing both dynamometers were repeated from the beginning. Records of torque (BIODEX) and force with torque calculation (DINABANG) were simultaneously obtained with two devices: the isokinetic BIODEX (Figure 2A) and lightweight portable DINABANG (Figure 2B).

**Figure 3:**
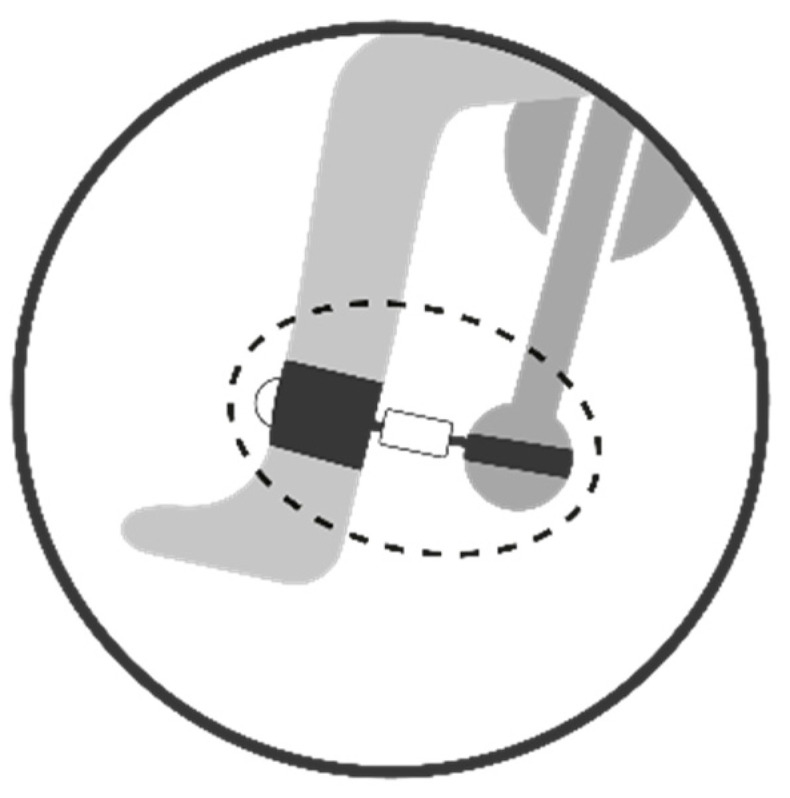
Representative image of the series configuration of the measuring instruments. The DINABANG connection (inside the dotted line) between the volunteer’s leg and the arm of the isokinetic machine ensures the simultaneous measurement of torque by both instruments.

The simultaneous measurement was carried out by connecting the two instruments in series. The DINABANG force measurement sensor was anchored at one end to the arm of the isokinetic machine and at the other end to the anklet on the patient’s leg. With this configuration, it was possible to measure the torque exerted by the volunteer with both instruments simultaneously (Figure 3).

The measurements were taken with subjects comfortably seated in BIODEX, with the trunk, hip and thigh stabilized (fixed by straps). To avoid compensatory movements, the subjects were instructed to grasp on the stabilization handles during the test. One of the authors orientated the knee extension axis (identified as a horizontal line passing through the femoral condyles) to coincide with the BIODEX axis of rotation. The hip and ankle joints were positioned at 90^o^. The strap of DINABANG was also affixed at the ankle of the leg being evaluated by BIODEX. DINABANG calculated the instant torque from force and chain orientation. Subjects were instructed to perform three repetitions of an MVIC of knee extensors, trying to maximally pull the chain for 3–4 s. A rest time of 90 s was given between each MVIC. Torque–time data during each repetition were obtained using a sampling frequency of 100 Hz.

### 2.3. Synchronization and Data Extraction

DINABANG and BIODEX torque–time curves were synchronized for each repetition a posteriori by the peak torque value of both time series, using Matlab R2023a (Natick, MA, USA; https://in.mathworks.com/ accessed on 12 May 2024). The precision of this synchronization is 100 Hz, i.e., 10 ms, which is the time between samples. DINABANG and BIODEX were connected in series so that any torque measured by one was coincident with the torque measured by the other.

### 2.4. Statistical Analysis

Paired comparisons of 38 MVICs of knee extensors were deemed sufficient (software G*Power, version 3.1.9.6) assuming an error probability of 0.05 [16]. Due to methodological reasons, one of the volunteers was excluded from the statistical analysis. From each of the 19 volunteers, 3 MVICs were obtained for each limb, resulting in 114 torque–time curves. A total of 114 paired MVICs and 22507 VICs (corresponding to torque–time curve data) of knee extensors were used for data analysis. A paired *t*-test was used to compare differences between the two instruments. Effect sizes (Cohen’s d) were interpreted with the following criteria: 0–0.19 trivial, 0.2–0.59 small, 0.6–1.19 moderate, 1.2–1.99 large, 2.0–3.99 very large, and >4.0 nearly perfect [17]. The Bland–Altman plot [18] was applied using open statistical software for the desktop and cloud (JAMOVI, macOS solid 2.3.28; www.jamovi.org accessed on 15 May 2024) according to the guidelines [19] to quantify the agreement between two quantitative measurements by determining the bias (or the mean of difference for normally distributed data and the median of the difference for non-normally distributed data) as a measure of accuracy and the limits of agreement as a measure of precision. The mean of the two measurements was plotted against the difference between them, with 95% of the differences expected to lie within the limits of agreement (±1.96 standard deviations from the mean and respective 95% confidence interval). The confidence interval of the bias illustrates the magnitude of the systematic error, while the confidence intervals of the limits of agreements provide an estimation of the extent of the possible sampling error [18,19]. An inspection of the slope of the linear regression (Bland–Altman plot) between both protocols (to check for proportional error) was performed. Intraclass correlation coefficients (ICCs) (model: one-way random) were calculated using IBM SPSS Statistics V.29 to quantify the consistency (repeatability) between BIODEX and DINABANG for each variable over a wide range of metabolic rates. Values less than 0.5, between 0.5 and 0.75, between 0.75 and 0.9, and greater than 0.90 were deemed indicative of poor, moderate, good, and excellent repeatability, respectively [20]. Statistical significance was established as *p* ≤ 0.05.

## 3. Results

We compared knee joint maximal isometric force–time-curve-related variables (MVICs and VICs) between the gold standard (isokinetic dynamometer) BIODEX and DINABANG by verifying the accuracy, precision, and repeatability. Table 1 shows mean and standard deviation of MVICs and comparison between the two devices (BIODEX vs. DINABANG). 

**Figure 4:**
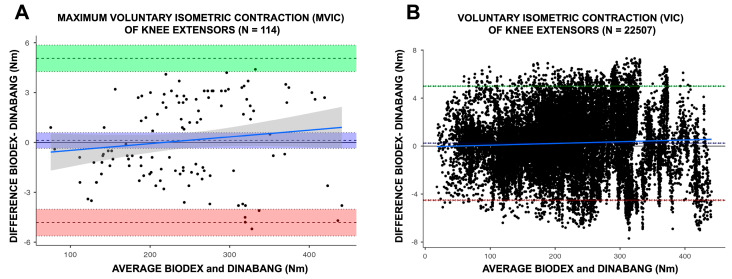
Bland–Altman plots comparing DINABANG with BIODEX. Plot of differences for (**A**) the maximum voluntary isometric contraction (MVIC) of knee extensors (Nm) and (**B**) voluntary isometric contraction (VIC) of knee extensors between BIODEX and DINABANG (Nm). Bias (dashed line), limits of agreement (dashed lines), and 95% CIs (shaded areas) between the BIODEX and DINABANG methods. Green, red, and blue represent the upper limit, lower limit, and bias, and the blue line represents the respective confidence bands and respective 95% CIs for the inspection of the slope of the linear regression between both conditions.

Regarding MVICs, we observed “excellent” repeatability (ICC: 1.00; 95%CI: 0.99 to 1.00; *p* < 0.001) between BIODEX (247.0 ± 79.5 Nm) and DINABANG (247.0 ± 74.8 Nm), and a “trivial” effect (mean diff.: 0.12 Nm (0.02%); 95%CI: −0.13 (−0.05) to 0.23 (0.09%) Nm; *p* = 0.606; d = 0.048) (Table 1). Bland–Altman plots revealed high accuracy for MVICs (bias: 0.12 Nm), with lines of equalities within the confidence interval of the mean differences. Additionally, we observed a consistent distribution (precision) inside the limits of agreement (−4.81 to 5.06 Nm) and respective 95% confidence interval, with no proportional error (*p* = 0.17) (Figure 4A).

Regarding voluntary isometric contractions (VICs), we observed “excellent” repeatability (ICC: 1.00; 95%CI: 0.99 to 1.00; *p* < 0.001) between BIODEX (219.0 ± 84.1 Nm) and DINABANG (218.0 ± 84.0 Nm) and a “trivial” effect (mean diff.: 0.24 Nm (0.11%); 95%CI: 0.08 (0.03%) to 0.11 (0.05%) Nm; *p* < 0.001; d = 0.100). Bland–Altman plots revealed a high accuracy for VICs (bias: 0.24 Nm), with lines of equalities within the confidence interval of the mean differences. Moreover, we observed a consistent distribution (precision) inside the limits of agreement (−4.5 to 4.9 Nm) and respective 95% confidence interval, with proportional error (*p* < 0.001) (Figure 4B).

Thus, these results indicate that DINABANG is an accurate, precise, and reliable device for torque measurement, providing portability for on-field evaluations.

## 4. Discussion

Quadricep strength is often assessed in sports due to its significance as an isometric strength parameter for monitoring training progression and detecting strength imbalances, which are predictors of injury risk [21]. Older adults with knee osteoarthritis commonly experience quadricep strength loss, increasing their risk of falls and serving as an indicator of sarcopenia [22,23]. Moreover, MVICs have been considered in some studies as a criterion for fatigue and recovery, facilitating the monitoring of training progress [24].

The aim of this study was to compare MVICs and paired VICs of knee extensors between the gold standard (isokinetic dynamometer) BIODEX and DINABANG, assessing their accuracy, precision, and repeatability. The main outcome suggests that DINABANG demonstrates excellent repeatability, consistent precision, and excellent accuracy for MVIC torque measurement compared to the BIODEX system, considered the gold standard. These results are particularly important and enhance the credibility for professionals involved in strength and conditioning, as well as rehabilitation, especially in situations that require portability for on-field assessments and/or the evaluation of a large number of patients within a short period of time. Despite that, our results indicated a tendency for increased differences between the values recorded by DINABANG and BIODEX when the force production was high. This may be attributed to the fact that subjects with a higher force output may have slightly flexed their knees before initiating the isometric exercise, thereby eliminating the pretension in DINABANG. To ensure the greatest accuracy in our results, all subjects completed a familiarization test day prior to participating in the study. Further research involving more trained subjects will be required to assess the influence of force production on DINABANG accuracy.

Unlike traditional muscular force sensors, the new system (DINABANG) allows the user to control the exact angle being tested due to the inclusion of an inertial movement unit. Although not fully validated, the system enables the analysis of force, torque, and mechanical power as a function of motion. Future applications may expand upon these findings by exploring various conditions, such as different training phases (competitive, pre-competitive, conditioning) or stages of rehabilitation, as well as different phases of intermittent games or medication regimens. The rationale behind this is that we anticipate that fatigue, overreaching, or even overtraining profiles may reveal valuable diagnostic information about training and rehabilitation loads that surpasses the insights gained from a single MVC reading.

We acknowledge some limitations to this study. Firstly, the power measurement capabilities of both instruments were not considered in the present research, which focused exclusively on the force and torque measurements of isometric contraction in normal volunteers. Secondly, studies involving individuals with movement disorders and aging populations may be valuable to expand the applicability of DINABANG, considering its varied force and coordination capabilities. Finally, DINABANG, compared to isokinetic machines, is not able to perform assessments at a controlled angular speed, unlike those performed by isokinetic machines. This limits its application in maximum strength assessments to the measurement of MVICs. In future research, the accuracy of DINABANG to assess another MVIC element would represent an interesting research proposal. However, DINABANG can measure joint angle in real time, which allows it to record data at different angles, giving greater details of an individual’s performance. It is important to highlight that it is essential to ensure an individual’s correct posture during assessments with DINABANG. It is important to have a seat with fasteners like those present on the BIODEX machine bench, which secure the individual to limit compensations during maximum strength assessments.

Our results suggest that sports medicine, training clubs, and rehabilitation facilities can assess neuromuscular function using a reliable device, enabling greater intervention control in real-life conditions. The minimum detectable difference typically reported for knee extensor torque is 25 to 28 Nm in the literature [25], which is approximately 100 times greater than the average difference between the BIODEX and DINABANG torque values. Consequently, DINABANG appears to be highly advantageous as a force recording tool in clinical trials within the health and performance field. DINABANG appears easily transportable for use in professionals’ offices, gyms, sports fields, and even athletes’ homes for remote follow-up. Since DINABANG is primarily a muscular power measurement system, we recommend that future studies compare force, torque, and mechanical power during movement.

## 5. Conclusions

DINABANG and BIODEX produced highly comparable MVIC and VIC responses across a wide range of values. We also noted highly accurate data with minimal bias. The distribution within each dataset and their respective 95% confidence intervals were consistently aligned for MVICs and VICs. The repeatability, as measured by the intraclass correlation coefficient (ICC), was excellent. Physiotherapists, support staff, and exercise and health professionals, as well as researchers, can now discern differences in values for strength capacities measured between the two devices.

## Figures and Tables

**Table 1:** Mean and standard deviation of maximal voluntary isometric contractions and comparison between the two devices (BIODEX vs. DINABANG).

	Right Lower Limb					
Serie	Repetition 1		Repetition 2		Repetition 3	
**System**	BIODEX	DINABANG	BIODEX	DINABANG	BIODEX	DINABANG
**Force (Nm)**	246.46 ± 75.66	246.06 ± 75.46	252.41 ± 73.58	252.84 ± 73.69	250.84 ± 73.69	250.58 ± 73.63
**Δ (Nm)**	0.40 ± 2.51		0.40 ± 2.51		0.26 ± 2.39	
**Sig.**	0.508		0.508		0.653	
	**Left Lower Limb**					
**Serie**	**Repetition 1**		**Repetition 2**		**Repetition 3**	
**System**	BIODEX	DINABANG	BIODEX	DINABANG	BIODEX	DINABANG
**Force (Nm)**	248.02 ± 88.06	248.23 ± 87.90	239.17 ± 82.93	239.41 ± 82.09	245.34 ± 79.43	245.22 ± 78.73
**Δ (Nm)**	0.22 ± 2.38		0.23 ± 2.57		0.12 ± 2.59	
**Sig.**	0.705		0.707		0.845	

## Data Availability

The data presented in this study are only available upon request from the corresponding author. The data are not publicly available as they contain information that could compromise the privacy of the study participants.
